# Public health round-up

**DOI:** 10.2471/BLT.16.010216

**Published:** 2016-02-01

**Meeting demand for the oral cholera vaccine**

The global oral cholera vaccine supply is set to double to 6 million doses this year after the World Health Organization (WHO) approved a third producer of the vaccine, Eubiologics Co., Ltd based in the Republic of Korea. Vaccine manufacturers must be approved under the WHO’s pre-qualification programme so that United Nations agencies can buy their vaccines. The photograph shows a 2015 cholera vaccination campaign in Malawi.

http://www.who.int/cholera/vaccines/double

**Figure:**
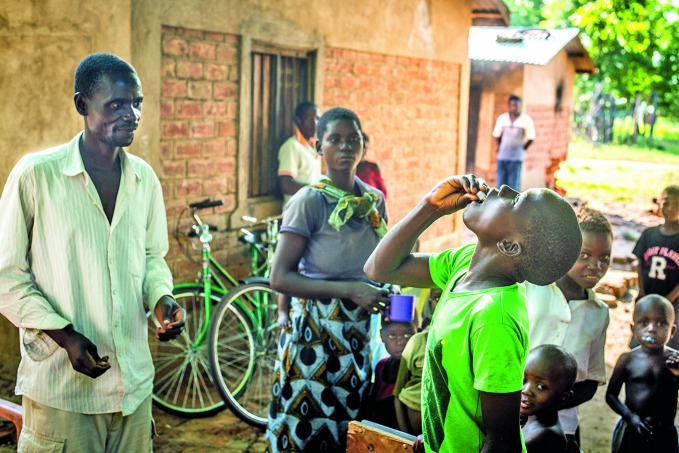
WHO/L Pezzoli

## Ebola transmission ends in Guinea 

Guinea is undergoing a period of heightened surveillance after WHO announced on 29 December that the Ebola virus was no longer being transmitted in the West African country.

If no Ebola cases are reported before the end of March, Guinea – where the outbreak began 2 years ago – can be considered free of the disease.

The Ebola outbreak in West Africa began in December 2013 in the south Guinean town of Guéckédou before spreading to neighbouring Liberia and Sierra Leone and, later, to seven other countries.

“This is the first time that all three countries – Guinea, Liberia and Sierra Leone – have stopped the original chains of transmission that were responsible for starting this devastating outbreak 2 years ago,” said Dr Matshidiso Moeti, WHO Regional Director for Africa. 

A country is considered free of human-to-human transmission of Ebola virus after 42 days or two incubation cycles of the Ebola virus since the last known case tested negative twice.

More than 2500 people died of Ebola during the outbreak in Guinea, in addition to about 9000 in Liberia and Sierra Leone. 

Since WHO announced the end of Ebola transmission in Liberia new cases have been reported as a result of the re-emergence of the virus persisting in a previously infected patient.

Screening for persistent virus among survivors is still needed as the microorganism may be present in the semen of some male survivors for as long as 12 months. 

WHO is currently working with the governments of the three West African countries to ensure that survivors receive adequate medical and psychosocial care. 

WHO announced the end of transmission of the virus in Sierra Leone in November. 

http://apps.who.int/ebola/current-situation/ebola-situation-report-6-january-2016

## More health workers needed 

Some countries in the WHO European Region may not have enough health workers to respond to the growing health needs of their ageing populations in coming years, according to a WHO report. 

The report, entitled *Core health indicators in the WHO European Region 2015,* with a special focus on human resources*,* shows that health workforce shortages and imbalances constitute a major public health concern for the region that requires prompt action. 

Although the number of health-care workers has increased overall in the region by nearly 10% over the last decade, this growth was uneven between countries and not necessarily in the countries where health professionals are most needed, the report said. 

One in three physicians is more than 55 years old, an increase of 6 percentage points over the past 7 years. 

The report found that there were five times more physicians in some countries than in others, and that some countries had nine times fewer nurses than others calculated per head of population. Greece registered 619 physicians per 100 000 population in 2013, for example, while Albania had 128. 

The number of medical graduates must increase to replace them, for example, in Albania, Bosnia and Herzegovina, Kyrgyzstan and Turkey, where the number of physicians per 100 000 habitants is below 200. In Albania, only 261 physicians graduated in 2013. 

The report is an annual publication providing an overview of the European health situation. The most recent edition provides key statistics on health workforce employment and education. 

The report also includes other key health statistics, such as mortality and morbidity data, demographic and socioeconomic indicators, health services utilization and health expenditure.

“Human resources are the cornerstone of the health system in any country, and the planning, regulation and management of the health workforce requires extensive intersectoral collaboration – both topics are at the heart of the Health 2020 policy,” says Dr Zsuzsanna Jakab, WHO Regional Director for Europe, referring to its core strategy. 

http://www.euro.who.int/en/data-and-evidence/core-health-indicators-in-the-who-european-region/core-health-indicators-in-the-who-european-region-2015.-special-focus-human-resources-for-health

## Medicines for Yemen 

WHO delivered more than 100 000 kilos of medical supplies and medicines to Taiz governorate in Yemen, but was unable to distribute them all because of continued fighting in spite of the 15 to 21 December ceasefire agreement. 

The supplies were destined for some 3 million people in need of humanitarian assistance in eight districts, including 392 000 internally displaced persons.

As of early January, drugs and medical supplies had been distributed to 13 hospitals and health centres and had helped to refill the contingency stock in Taiz. 

These provisions included oxygen cylinders, surgical devices and trauma management equipment. 

But WHO was unable to distribute 22 000 kilos of medical aid to health facilities in three districts of Taiz City, where 400 000 people were in desperate need of items such as oxygen cylinders, fluids and consumables for kidney dialysis. 

“WHO is deeply concerned about the continuous lack of humanitarian access to Taiz City, depriving people of basic health care and violating their essential human rights,” said Dr Ahmed Shadoul, WHO Representative in Yemen. 

“WHO re-emphasizes the crucial need for uninterrupted delivery of health services and calls upon all concerned parties to respect the basic rights of all Yemenis to access health-care services,” Shadoul said. 

http://www.emro.who.int/media/news/who-calls-for-immediate-access-to-taiz-city-for-delivery-of-life-saving-health-supplies.html

**Cover photo**

Somali refugees leaving Shimbiro Beach to board smugglers’ boats to Yemen. The photograph was shortlisted for the Prix Pictet photo competition in 2015. It is from a collection entitled *A million shillings: escape from Somalia* by London-based photographer Alixandra Fazzina. The competition was sponsored by the United Nations refugee agency and the International Organization for Migration.

**Figure:**
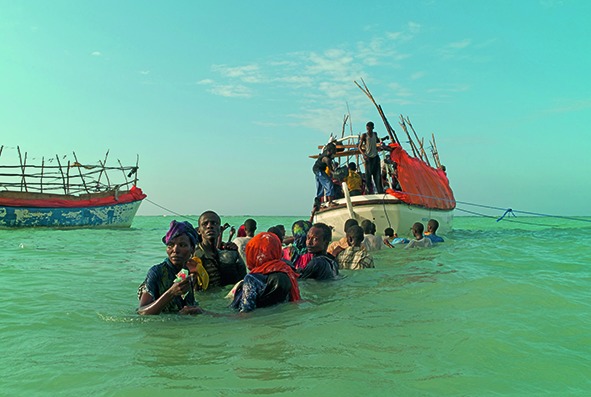
Alixandra Fazzina ©Prix Pictet Ltd 2015

## Smoke-free policies 

Indoor smoking bans to protect non-smokers from exposure to tobacco smoke have won broad public support in nine European countries, according to a WHO policy brief released last month. 

The 20-page policy brief entitled *How can we best protect non-smokers from exposure to tobacco smoke?* reviewed surveys and studies in Albania, Bulgaria, Greece, Ireland, Malta, Spain, Turkey, Turkmenistan and the United Kingdom of Great Britain and Northern Ireland. 

It found that strong public support for comprehensive smoke-free policies in these countries had led to a high level of enforcement – and compliance with – such policies.

Smoke-free policies include laws or health and safety regulations that ban smoking in indoor public places such as offices, restaurants and bars as well as buses and trains.

The WHO policy brief noted that such measures had resulted in considerable savings for the health systems of these countries and concluded that smoke-free measures do not deter tourism or result in economic losses for government budgets since tobacco taxes can be increased. 

“Smoke-free legislation works, but it is of key importance that certain indicators are not measured prematurely. Doing so would raise the risk of incorrectly portraying low levels of impact and, thus, of jeopardizing political support of the policy,” the policy brief says, adding that only countries that had implemented smoke-free laws for at least 2 years were evaluated.

There is no safe level of exposure to second-hand smoke, the policy brief notes, adding that comprehensive smoke-free laws are the only effective means of eliminating the risks associated with passive smoking. 

http://www.euro.who.int/__data/assets/pdf_file/0005/276557/How-can-we-best-protect-non-smokers,-Evidence-Brief-Eng.pdf?ua=1

## Neglected tropical diseases 

A new neglected tropical diseases project began in Africa last month, aiming to eliminate five diseases – onchocerciasis, lymphatic filariasis, trachoma, schistosomiasis and soil-transmitted intestinal worms – by 2020, through mass drug administration. 

The Expanded Special Project for Elimination of Neglected Tropical Diseases (ESPEN) is the new technical arm of the Neglected Tropical Diseases Programme and will provide technical support to endemic countries in their efforts to meet the 2020 elimination target. 

This technical support includes the prevention and management of disabilities, such as blindness, with the overall goal of alleviating poverty and raising productivity and quality of life for the communities affected. 

The US$ 10 million project is hosted by the WHO Regional Office for Africa and takes over from the African Programme for Onchocerciasis Control that was established in 1995 and ended last year.

It is the result of a consensus reached by endemic countries, neglected tropical disease stakeholders and WHO at consultative meetings in April and July 2015, and will be officially launched in May.

http://www.afro.who.int/en/media-centre/pressreleases/item/8239-the-apoc-closes-and-a-new-body-set-up-to-eliminate-neglected-tropical-diseases.html

**Looking ahead**

**25–26 February – Ministerial Conference on Immunization in Africa**, in Addis Ababa, Ethiopia. http://immunizationinafrica2016.org
**24 March – World Tuberculosis Day.**
**7 April – World Health Day**. The 2016 theme is diabetes.
**24–30 April – World Immunization Week**.
**23–28 May – Sixty-ninth World Health Assembly**, Geneva, Switzerland.

